# The impact of professional role and demographic characteristics on job satisfaction and retention among healthcare professionals in a military hospital

**DOI:** 10.1111/nuf.12777

**Published:** 2022-07-09

**Authors:** Sherita House, Jaime Crandell, Melissa Miller, Christopher Stucky

**Affiliations:** ^1^ Indiana University School of Nursing Indianapolis Indiana USA; ^2^ University of North Carolina at Chapel Hill School of Nursing Chapel Hill North Carolina USA; ^3^ Center for Nursing Science and Clinical Inquiry (CNSCI) Womack Army Medical Center Fort Bragg North Carolina USA; ^4^ Center for Nursing Science and Clinical Inquiry (CNSCI) Landstuhl Regional Medical Center Landstuhl Germany

**Keywords:** job satisfaction, military health, quantitative, research, retention

## Abstract

**Background:**

Job satisfaction is significantly associated with retention. Although several factors are associated with job satisfaction and retention (pay, leadership, mentorship), the association of demographic characteristics has been understudied in the literature.

**Purpose:**

To explore whether professional role and demographic characteristics are associated with job satisfaction and intent to stay among nurses and physicians in a military medical center.

**Methods:**

We conducted a descriptive, exploratory, cross‐sectional study, and collected data via surveys. We used multiple regression to evaluate study variables.

**Results:**

Two hundred and eighty‐nine participants completed the survey. Professional role and demographic characteristics were not associated with job satisfaction. Professional role, race, and education were associated with intent to stay for military respondents. Physicians (*β *= 0.53, *p* = .0259) and Caucasians (*β* = −0.55, *p* = .0172) reported lower intent to stay; respondents with graduate degrees reported higher intent to stay (*β* = 2.47, *p* = .0045). Professional role and demographic characteristics were not associated with intent to stay for civilians.

**Conclusion:**

Job satisfaction and retention of nurses and physicians are critical to the quality of care. Civilian and military healthcare leaders should focus on interventions that enhance job satisfaction and retention as a strategy to improve patient and staff outcomes alike.

## 1 INTRODUCTION

Job satisfaction and retention among nurses and physicians are significantly associated with healthcare quality and safety.^1^ Job satisfaction is the degree to which employees enjoy their job,^2^ and retention is the ability of an organization to retain its employees.^3^ The determinants of job satisfaction and retention are multifactorial and complex, which poses challenges to healthcare leaders who seek to develop initiatives to enhance job satisfaction and retention. The Military Health System is an important setting to explore job satisfaction and retention because of the unique roles and mission requirements of the nurses and physicians who work in these facilities.^4^, ^5^, ^6^ Civilian nurses and physicians who work in military treatment facilities supplement staffing and support operational readiness when active‐duty military nurses and physicians deploy in wartime crises or humanitarian efforts^5^; therefore, retaining civilian nurses and physicians is important to the quality of care. Military nurses and physicians engage in rigorous and time‐consuming training to complete occupational and specialty requirements for leadership development. Thus, the Military Health System places a high value on retaining nurses and physicians, as their training requirements are more costly and robust than their civilian counterparts.^7^ It may also be more challenging to replace seasoned military nurses and physicians who have the unique experience of providing care to patients on battlefields or austere environments. The retention of both military and civilian nurses and physicians who work in military medical centers may make the difference between life and death, especially during wartime.

Although several factors (e.g., burnout, pay, leadership support, high‐quality communication, and relationships between nurses and physicians) are associated with job satisfaction among nurses and physicians,^8^, ^9^, ^10^, ^11^, ^12^, ^13^ less attention has been given to how demographic characteristics influence job satisfaction. Most researchers report the demographic characteristics of their sample; however, there is a gap in the literature regarding whether demographic characteristics are associated with job satisfaction. A few researchers have explored if demographic characteristics are related to job satisfaction. However, the results of these studies are mixed. For example, Carthon et al.^14^ found that Black nurses in civilian community‐based settings reported higher job dissatisfaction than Caucasian nurses. Other researchers have reported similar findings in civilian healthcare settings; in these studies, minority nurses experienced lower job satisfaction.^15^, ^16^ In other studies, however, race,^17^ age,^18^, ^19^ and gender^20^, ^21^ were not associated with job satisfaction.

Researchers have also reported mixed results regarding relationships between demographic characteristics and retention. In some studies, race,^14^ age,^22^, ^23^ and gender^24^ were associated with retention; in other studies, these demographic characteristics were not associated with retention.^25^, ^26^ Due to the lack of consensus in the current literature, we identified two significant knowledge gaps related to demographic characteristics, job satisfaction, and retention: (1) there is limited research about job satisfaction and retention among healthcare professionals in military medical centers; (2) little is known regarding whether professional roles (e.g., nurse or physician) and demographic characteristics (e.g., race, age, sex, and rank) are associated with job satisfaction and retention among civilian and military healthcare professionals.

Exploring the relationships among professional roles, demographic characteristics, job satisfaction, and retention in civilian and military healthcare settings is important for various reasons. First, race, age, and sex diversity are increasing in the nursing and medical workforces.^27^, ^28^, ^29^, ^30^ The nursing workforce was once predominantly Caucasian, young, and female, while the physician workforce was predominately Caucasian and male.^27^, ^29^, ^30^ Over the last two decades, more minorities have joined the nursing and physician workforce, there is an increase in male nurses and female physicians, and a variety of generational cohorts exist in the nursing and medical professions.^27^, ^28^, ^29^, ^30^

Second, the military has a formal ranking structure with clear lines of authority among military service members.^31^, ^32^, ^33^ Thus, to provide care, nurses and physicians must navigate a complex web of rank and authority gradients predicated by military service and professional roles. Rank diversity is another unique type of diversity found in military medical centers; enlisted service members typically serve in paraprofessional roles, such as medics, corpsmen, or licensed practical nurses (LPNs), while registered nurses (RNs) and physicians are commissioned officers. There is also a ranking structure among civilian nurses and physicians who work as government service employees. General Schedule (GS) employees are professionals who work for the federal government and their ranking system consists of 15 grades, from GS‐1 (the lowest level) to GS‐15 (the highest level).^34^ This clear ranking structure sometimes allows nurses to hold a higher GS grade than their physician counterparts, and vice versa. However, no matter the rank or grade of the employee, they are expected to work collaboratively with others to provide patient care. Examining relationships among rank, job satisfaction, and retention is critical to improving work processes among members of different ranks.

A third reason to explore relationships among professional roles, demographic characteristics, job satisfaction, and retention is that individuals create relationships with people who are similar to themselves.^35^, ^36^ Additionally, individuals who perform similar tasks within the same professional role are less likely to create relationships with professionals who perform dissimilar tasks.^37^ For example, Mascia et al.^37^ found that physicians were more likely to create relationships with physicians who shared similar professional backgrounds in terms of their field of specialization. Given that people create relationships with people who are similar to themselves^35^ and high‐quality relationships and communication are associated with higher job satisfaction and retention,^38^, ^39^, ^40^ it is important to explore if professional role and demographic characteristics influence job satisfaction and retention. Finally, job satisfaction is positively associated with retention,^41^, ^42^, ^43^, ^44^ and dissatisfied nurses and physicians are more likely to resign or relocate.^41^ Decreased retention ultimately affects patient outcomes and operational readiness in military hospitals.^5^

To our knowledge, researchers have not extensively explored if professional role and demographic characteristics are associated with job satisfaction and retention in a military hospital context. Thus, we conducted a descriptive, exploratory, cross‐sectional survey study to explore job satisfaction and intent to stay among Army and civilian nurses, resident physicians, and physicians working in a military medical center. The purpose of our study was to determine if professional role and demographic characteristics are associated with job satisfaction and intent to stay. We asked the following research question: Are demographic characteristics (race, age, sex, rank) associated with job satisfaction and intent to stay for Army and civilian nurses, resident physicians, and physicians? Our study lacked a hypothesis because we desired to explore the associations among professional roles, demographic characteristics, job satisfaction, and intent to stay to develop targeted hypotheses for future research.

## 2 METHODS

### 2.1 Design and sample

We conducted a descriptive, exploratory, cross‐sectional survey study on 12 patient care units (e.g., emergency department, operating room, labor and delivery, neonatal intensive care, postanesthesia care unit, medical‐surgical, intensive care, intermediate care, behavioral health, medical telemetry, women and newborn care, urgent care) in a 138‐bed military medical center in the southeastern United States. We invited a convenience sample of active‐duty and civilian LPNs, RNs, resident physicians, and physicians to complete a survey regarding their experiences of job satisfaction and intent to stay. Our inclusion criteria were: (1) 18 years of age or older; (2) employed at least 3 months at the study site; (3) employed as a military or civilian RN, or LPN; (4) military or civilian physician; (5) military or civilian resident physician, and (6) English language literacy. Exclusion criteria included: (1) nursing assistants; (2) nurse practitioners; (3) physician assistants; and (4) nurse‐midwives. We excluded these groups at the request of the hospital research council to limit the overall staff burden, given that several research studies had been previously conducted at the study site. We selected nurses (LPNs and RNs) and physicians (resident physicians and physicians) because they comprise the largest portion of the healthcare workforce.^27^, ^30^

### 2.2 Recruitment activities and data collection

Recruitment activities began 1 month before data collection and included face‐to‐face meetings with eligible participants. We sent a detailed email to eligible participants explaining the purpose of the study. Data were collected from January 2020 to March 2020. We implemented Dillman's Total Design Method^45^ to enhance response rates and sent three reminder notices to eligible participants via email. Participants had the option to complete the survey electronically or via hard copy. Completing and submitting the survey implied consent to participate in the study. Firewall protection issues at the study site prevented some participants from receiving the survey via email. Thus, we provided a second option for participants to complete the survey via hard copy. The research team placed hard copies of the survey in a folder and a locked storage box in a designated area on each unit for participants completing hard copy surveys to submit and store their data securely. We collected the surveys weekly during data collection.

### 2.3 Measures

We combined three tools (relational coordination, job satisfaction, and intent to stay) into one 47‐item survey. See Supporting Information: File 1 to review the participant survey. Relational coordination defined as, “a mutually reinforcing process of interaction carried out for the purpose of task integration,”^46^ ^, p. 301^ is a validated tool.^46^ Relational coordination encompasses four communication dimensions (frequent, timely, accurate, problem‐solving) and three relationship dimensions (shared goals, shared knowledge, and mutual respect).^47^ We explored relational coordination because high‐quality communication and relationships are associated with higher job satisfaction and retention.^10^, ^11^, ^12^, ^13^ However, this manuscript focuses on the data analysis concerning job satisfaction and intent to stay.

#### 2.3.1 Independent variables

Respondents self‐reported the demographic variables in our study (race/ethnicity, age, sex, rank, education, and experience). (see Table 1).

**Table 1:** Demographic variable descriptions and study instruments

Variable	Level of measurement			Variable description
Race/ethnicity	Categorical			Black, Caucasian, Other
Age	Continuous			Reported in years
Sex	Nominal			Female or male
Rank	Ordinal			Officers: O‐1, O‐2, O‐3, O‐4, O‐5, O‐6 Enlisted: E‐1, E‐2, E‐3, E‐4, E‐5, E–6 civilians
Experience	Continuous			The number of years the participant has been in their profession
Education	Categorical			The highest degree obtained (diploma, associate, baccalaureate, or graduate degree)

Study instruments				
Instrument	Number of items	Response format	*α*	Validity and factor analysis
Job satisfaction	3	1 single‐item measure 2 open ended questions	‐	‐
Intent to stay	4	1 = “strongly disagree,” 2 = “disagree,” 3 = “neither agree nor disagree,” 4 = “agree,” 5 = “strongly agree.”	.85	Convergent & discriminant exploratory factor analysis

#### 2.3.2 Dependent variables

##### Job satisfaction

Job satisfaction is the extent to which people enjoy their job.^2^ We measured job satisfaction with a single‐item question because single‐item measures of job satisfaction strongly correlate with multiple‐item scales.^48^, ^49^ We developed a one‐item job satisfaction question based on our literature review and expert opinion to satisfy the research question. We piloted the question with a small group of personnel to test for validity and reliability and to assess question clarity and sensitivity, allowing for revisions based on feedback. We asked participants, “On the whole, how satisfied are you with your present job?” Responses ranged from “1 = very dissatisfied” to “5 = very satisfied,” with higher scores indicating better job satisfaction.

##### Intent to stay

We conducted a thorough review of the literature to determine how to measure retention and which instrument to use for this study. Retention was measured using a four‐item intent to stay scale. Intent to stay is the extent to which employees plan to continue membership with their employer.^2^ This instrument was developed by Kim et al.^2^ in a study exploring intent to stay among physicians in an Air Force hospital. This instrument has good psychometric properties, with a Cronbach's *α* of .85.^2^ Researchers have used this scale to explore intent to stay among nurses and physicians in both military and civilian hospitals. Two nurse scientists on the research council at the study site reviewed the intent to stay survey questions developed by Kim et al.^2^ and agreed that this scale measured what we purported to measure among military and civilian respondents in our study. The Cronbach's *α* for our study was .85 for the civilian intent to stay scale and .91 for the military intent to stay scale. Sample items were: “I will be reluctant to leave this hospital” for the civilian intent to stay scale and “I will be reluctant to leave the Army” for the military intent to stay scale. Responses ranged from “1 = strongly agree” to “5 = strongly disagree.” We used reverse scoring where appropriate so that higher scale scores indicate higher intent to stay.

### 2.4 Data analysis

We used *t*‐tests and one‐way analysis of variance to explore bivariate relationships between the independent variables (demographic variables) and outcome variables (job satisfaction and intent to stay). We organized data by professional role, employment status (military or civilian), and unit type (e.g., intensive care, labor and delivery, medical telemetry). We used multiple regression to explore whether professional role and demographic characteristics predicted job satisfaction and intent to stay, including all nonoutcome variables (professional roles and demographic characteristics) as predictors. For analytic purposes, we combined the race/ethnicity variable (Black/African American, Caucasian, and Other) and the rank variable (civilian, enlisted, and officer) into three groups. Eighty‐seven participants chose to complete the survey electronically, and 202 participants completed the survey via hard copy. For the analyses, we used Stata statistical software (Stata Statistical Software, Version 16. College Station, TX: Stata Corp LLC).^50^

### 2.5 Statistical power

We conducted a power analysis before beginning data collection. We fit regression models that included all participants (estimated *N* = 672). Our sample size had at least 80% power to detect an effect of *d* = 0.22 for a dichotomous variable (e.g., sex) and a partial correlation of 0.12 for a continuous variable (e.g., age). This large sample is more than adequate for the exploration of the associations between demographic characteristics, job satisfaction, and intent to stay.

## 3 RESULTS

### 3.1 Descriptive statistics

In our study, 289 healthcare professionals completed the survey, yielding a 43% response rate. Sixty‐one percent of respondents identified as Caucasian, 18% identified as Black, and 21% identified as Other. Most respondents were nurses (75%), females (78%), and civilians (70%). Forty‐nine percent of respondents had a Baccalaureate degree (*n* = 140). The mean years of experience was 11.9 years (SD = 9.5 years). See Table 2.

**Table 2:** Descriptive statistics for study variables

	Entire sample (*n* = 289)		Military (*n* = 86)		Civilians (*n* = 203)	
Variable	*n* or Mean	% or SD	*n* or Mean	% or SD	*n* or Mean	% or SD
Professional role						
LPN	26	10%	16	19%	10	5%
RN	217	75%	37	43%	180	89%
Resident	10	3%	10	11%	N/a	N/a
Physician	36	12%	23	27%	13	6%
Race/ethnicity						
Black	51	18%	7	8%	44	22%
Caucasian	171	61%	58	69%	113	57%
Other	60	21%	19	23%	41	21%
Sex						
Female	216	78%	43	54%	173	88%
Male	60	22%	36	46%	24	12%
Military rank						
Officer	68	24%	68	79%	N/a	N/a
Enlisted	18	6%	18	21%	N/a	N/a
Civilian	203	70%	N/a	N/a	203	70%
Education						
Diploma	17	6%	10	12%	7	3%
Associate	60	21%	7	8%	53	27%
Baccalaureate	140	49%	31	36%	109	55%
Graduate	67	24%	38	44%	29	15%
Experience (years)	11.9	9.5	5.15	5.52	14.8	9.40
Age (years)	40.0	11.7	31.29	8.88	44.22	10.51
Job satisfaction	3.72	1.04	3.49	0.92	3.81	1.07
Intent to stay	2.39	1.11	2.39	1.11	3.91	1.00

*Note*: “Other” race/ethnicity variable = (Asians, Hispanics, Native Americans, and participants who identified their race/ethnicity as other).

Abbreviations: LPN, licensed practical nurses; N/a, not applicable; RN; registered nurses; SD, standard deviation.

### 3.2 Bivariate analysis

Job satisfaction varied across professional roles (*p* = .014); RNs reported the highest job satisfaction and resident physicians reported the lowest. Civilians reported the highest mean job satisfaction, followed by enlisted, and officers (*p* = .035). Professional role and education were associated with intent to stay for military respondents. Military resident physicians reported the lowest intent to stay (*p* = .007), and participants with a baccalaureate degree reported the highest intent to stay (*p* = .012). Intent to stay did not vary across professional role for civilian respondents (see Table 3). Job satisfaction was positively associated with intent to stay for both civilian (*r* = 0.48, *p* = .000) and military respondents *(r* = 0.28, *p* = .008). (see Table 4).

**Table 3:** Bivariate analysis

	Job satisfaction Mean (SD)	*p*	Civilian intent to stay Mean (SD)	*p*	Military intent to stay Mean (SD)	*p*
Professional role						
LPN	3.65 (1.13)	**.014**	3.86 (0.74)	.453	1.94 (0.99)	**.007**
RN	3.80 (0.98)		3.94 (1.00)		2.84 (0.95)	
Resident	2.8 (0.79)		N/a		1.93 (0.81)	
Physician	3.53 (1.21)		3.58 (1.19)		2.16 (1.31)	
Race/ethnicity						
Black/African American	3.78 (1.10)	.956	3.85 (0.93)	.714	2.36 (1.53)	.386
Caucasian	3.75 (0.98)		3.91 (1.04)		2.32 (0.97)	
Other	3.72 (1.08)		4.03 (1.03)		2.72 (1.33)	
Sex						
Female	3.77 (0.07)	.953	3.93 (0.08)	.469	2.40 (0.14)	.729
Male	3.78 (0.14)		3.77 (0.22)		2.49 (0.21)	
Rank						
Enlisted	3.44 (0.94)	**.035**	N/a	.194	2.47 (1.08)	.194
Officer	3.67 (0.84)		N/a		2.08 (1.19)	
Civilian	3.81 (1.07)		3.91 (1.00)		N/a	
Education						
Diploma	3.88 (1.05)	.062	3.96 (0.77)	.046	1.55 (0.95)	**.012**
Associate	3.85 (1.11)		4.17 (0.89)		2.61 (1.31)	
Baccalaureate	3.78 (0.92)		4.00 (1.00)		2.79 (0.87)	
Graduate	3.42 (1.12)		3.52 (1.14)		2.24 (1.17)	

*Note*: The “Other” category included Asians, Hispanics, and Native Americans. Bolded font denotes significance.

Abbreviations: LPNs, licensed practical nurses; SD, standard deviation; RNs, registered nurses; N/a, not applicable.

**Table 4:** Correlation matrix

	Job satisfaction	Intent to stay (civilian)	Intent to stay (military)	Age	Experience
Job satisfaction	1.00				
Intent to stay (civilian)	**0.48** **	1.00			
Intent to stay (military)	**0.28** *		1.00		
Age	0.02	−0.06	0.06	1.00	
Experience	0.11	−0.06	0.04	**0.79** **	1.00

*Note*: Bold denotes significance.

** *p* < .0001

* *p* < .01.

### 3.3 Regression analysis: Job satisfaction and intent to stay

Table 5 presents the results of multiple regression analyses to examine if professional role and demographic characteristics are associated with job satisfaction and intent to stay adjusting for covariates. *R* ^2^ ranged from 0.06 to 0.35 for the job satisfaction and intent to stay models. Professional role and demographic characteristics were not significant predictors of job satisfaction. Professional role, race/ethnicity, and education were statistically significant predictors of intent to stay for military respondents. Physicians (*β* = −0.53, *p* = .026) and Caucasians (*β* = −0.55, *p* = .017) in the military reported lower intent to stay, and military respondents with graduate degrees reported the highest intent to stay (*β* = 2.47, *p* = .0045). Professional role and demographic characteristics were not significant predictors of intent to stay for civilian respondents. Age, experience, and rank were not associated with job satisfaction or intent to stay (see Table 5).

**Table 5:** Regression analysis: Job satisfaction and intent to stay

	Job satisfaction (*n* = 258)		Civilian intent to stay (*n* = 183)		Military intent to stay (*n* = 77)	
	*β* (SE)	*p*	*β* (SE)	*p*	*β* (SE)	*p*
Professional role (Reference group LPNs)						
RNs	−0.038 (0.401)	.313	−0.041 (1.029)	.998	1.055 (0.637)	**.026**
Residents	−0.877 (0.602)		‐		−0.398 (0.861)	
Physicians	−0.266 (0.511)		−0.020 (1.130)		−0.531 (0.827)	
Race/ethnicity (Reference group Black/African American)						
Caucasian	0.005 (0.170)	.78	0.039 (0.186)	.465	−0.552 (0.464)	**.017**
Other	0.121 (0.212)		0.280 (0.245)		0.318 (0.511)	
Age	−0.014 (0.001)	.140	−0.015 (0.011)	.170	−0.012 (0.029)	.691
Sex (Reference group males)						
Females	−0.250 (0.189)	.186	−0.018 (0.284)	.949	−0.412 (0.259)	.116
Rank (Reference group officers)						
Enlisted	−0.249 (0.407)	.511	N/a		0.870 (0.960)	.368
Civilian	0.16 (0.20)		N/a		N/a	
Experience	0.014 (0.011)	.200	0.006 (0.012)	.624	0.041 (0.041)	.32
Education (Reference group diploma)						
Associate	0.109 (0.391)	.342	0.371 (1.086)	.090	1.705 (0.541)	**.005**
Baccalaureate	−0.189 (0.401)		−0.026 (1.096)		1.630 (0.827)	
Graduate	−0.233 (0.445)		−0.248 (1.129)		2.471 (0.900)	
R^2^	0.07		0.06		0.35	

*Note*: The “Other” category included Asians, Hispanics, and Native Americans. Bolded font denotes significance.

Abbreviations: LPNs, licensed practical nurses; N/a, not applicable; Ref, reference group; RNs, registered nurses.

## 4 DISCUSSION

In this study, we explored if professional roles and demographic characteristics were associated with job satisfaction and intent to stay among nurses, resident physicians, and physicians in a military medical center. We found that demographic characteristics (race/ethnicity, age, sex, and rank) and professional role (LPN, RN, resident physician, physician) were not associated with job satisfaction. Although findings regarding the associations between demographic characteristics and job satisfaction are mixed, our results are similar to previous researchers who reported that race,^17^ age,^18^, ^19^ and gender^20^, ^21^ were not related to job satisfaction. We attribute our results to the Army's mission‐first culture. Underpinning the performance of military healthcare teams are cultural aspects in which a focus on the mission permeates its members' attitudes and work practices.^51^ In our setting, the mission was patient care, and the participants potentially felt a heightened sense of professional duty to provide healthcare to military service members and their families. Consequently, we believe that demographic characteristics and professional roles were less important to the job satisfaction of our participants because their mission was to provide high‐quality patient care.

Professional role, race/ethnicity, and education were associated with intent to stay for military respondents. In our study, military physicians reported lower intent to stay, and military nurses reported higher intent to stay. Military physicians make substantially less than their civilian counterparts, which may affect retention when their service obligation ends.^52^ Military physicians also have less autonomy than civilian physicians, and physicians may be less likely to stay if they cannot practice in their specialty.^52^, ^53^ For example, military physicians may not have the autonomy to choose their specialty practice, as these decisions are based on the needs of their service branch. For physicians, the decision to stay in the military may mean decreased pay, reduced autonomy, and long periods away from family due to training and deployments.^51^ Unlike military physicians, military nurses reported higher intent to stay. Civilian nurses' intent to stay was nonsignificant. Prior researchers have focused on retaining military nurses in military treatment facilities. Yet, civilian nurses supplement staffing when active‐duty nurses deploy. Given the results of our study, we believe that military hospital leaders should seek opportunities to better understand factors that are associated with the intent to stay of civilian nurses working in military treatment facilities.

Race/ethnicity was also associated with intent to stay for military respondents, with Caucasians reporting lower intent to stay. Researchers have explored race and retention in civilian healthcare settings; however, these studies had mixed findings. For example, in one study, Black nurses were less likely to stay compared to Asian and Caucasian nurses.^15^ Carthon et al.^14^ reported similar results; in their study, Black nurses were more likely to report job dissatisfaction and intent to leave compared to Caucasian nurses. In another study, race was not a predictor to leave among male nurses.^54^ Caucasian respondents in this study may perceive that they have better career opportunities in other healthcare facilities. For example, military physicians and resident physicians were predominately Caucasian in our sample, and they also reported lower intent to stay. Moreover, the study site was a level three trauma center, which may influence the retention of nurses and physicians seeking a more challenging level of care, expanded specialties, and higher patient acuity.

Education was also significantly associated with intent to stay. Military respondents with graduate degrees reported the highest intent to stay scores. In previous studies, education level was associated with intent to stay. For example, previous researchers have reported that nurses' education level is associated with turnover as an antithesis to intent to stay and is often operationalized as nurses possessing a baccalaureate degree.^55^ Active‐duty service members achieve higher levels of education and advanced clinical licensure earlier than their civilian counterparts, and officers are required to have a bachelor's degree at entry.^4^ Both military and civilian nurses and physicians are required to meet specific educational milestones for career advancement. Therefore, junior nurses and physicians may report lower intent to stay due to military and civilian education and training requirements for degree and licensure advancement.

In our study, professional role and demographic characteristics were not significant predictors of intent to stay for civilian respondents. Our findings are dissimilar to researchers who report that race,^14^ age,^22^, ^23^ and gender^24^ are associated with retention. For example, Blegen et al.^22^ found that younger nurses reported higher intent to stay than older nurses, which is contrary to the results of Ramoo et al.,^56^ who found that younger nurses were more likely to leave the organization within 2 years of employment. We also found that rank was not associated with intent to stay for civilian respondents. The unique rank structure of government service civilian personnel in the Military Health System may have influenced civilian responses in our study. Unlike military service members, government service civilians are not addressed by their rank. Therefore, civilians may not identify with their rank beyond acknowledging rank as a pay grade.

### 4.1 Recommendations for hospital leaders

We recommend that hospital leaders implement evidence‐based, modifiable practices and policies to enhance job satisfaction and retention, such as formal mentorship programs,^56^, ^57^, ^58^ recognition and rewards,^59^ professional development programs,^60^ increased opportunities for advancement,^14^ and increased salary and benefit packages.^14^, ^61^ We also recommend that hospital leaders make changes at the organization level by using surveys to assess diversity, inclusion, and instances of discrimination; these surveys should be followed by measures to address identified issues and change the organizational culture.^62^

Given that nurses are the largest healthcare professionals in the healthcare sector, nurse managers should focus on interventions to improve staff nurses' job satisfaction and intent to stay by enhancing nurses' autonomy and implementing strategies to improve communication. Nurse managers can enhance nurses' autonomy by including staff nurses in decision‐making that affects nursing practice and allow staff nurses to have more control over their work.^63^ Nurse managers can improve communication in their unit by implementing relational work practices such as relational coordination.^64^ We also suggest that nurse managers use mechanisms such as one‐on‐one meetings, focus group discussion sessions, and suggestion boxes to enhance communication and feedback between nurse managers and staff nurses.^63^ These interventions are a cost‐effective way to improve nurses' job satisfaction and intent to stay.

### 4.2 Limitations

We conducted this study at a single Army medical center, and the generalizability of the findings may not be applicable in all settings, including other branches of service (e.g., Air Force and Navy hospitals), larger military hospitals, or civilian hospitals. The determinants of job satisfaction and retention are complex. Although we addressed a gap in knowledge concerning the impact of demographic characteristics on job satisfaction and retention, our results are not exhaustive or comprehensive. A more comprehensive research approach would include thoroughly investigating how demographic characteristics and professional roles moderate or mediate job satisfaction and retention to better understand how demographic characteristics impact job satisfaction and retention. However, our results are essential for researchers to understand concepts clearly. Lastly, we only collected data from LPNs, RNs, resident physicians, and physicians. Future researchers should explore job satisfaction and intent to stay from the perspective of other nursing professionals, such as nurse practitioners and clinical nurse specialists to gain a richer understanding of job satisfaction and intent to stay among nurses.

### 4.3 Directions for future research

Future directions for research include exploring the demographic variables in this study as moderating variables rather than predictor variables. For example, the respondents in this study were 75% female, and females usually report higher job satisfaction than males.^65^, ^66^ Sex was not a significant predictive variable in our study; however, sex could be explored as a moderating variable in future studies. Race could also be explored as a moderating variable given that individuals create relationships with individuals who are similar to themselves,^35^, ^36^ and high‐quality relationships are significantly associated with higher job satisfaction and retention.^38^, ^39^, ^40^, ^41^ Exploring demographic characteristics as moderating variables could direct interventions for future research and help hospital leaders improve work environments, construct retention strategies, and create recruitment measures that attract a diverse group of nurses and physicians.

## 5 CONCLUSION

This manuscript explores the relationships among professional roles, demographic characteristics, job satisfaction, and intent to stay among nurses and physicians working in a military medical center. We found that RNs reported the highest levels of job satisfaction, and job satisfaction was positively associated with intent to stay for both civilian and military respondents. Demographic characteristics (race/ethnicity, age, sex, and rank) and professional role (LPN, RN, resident physicians, physician) were not associated with job satisfaction, while professional role, race/ethnicity, and education significantly predicted intent to stay for military respondents.

## CONFLICT OF INTEREST

The authors declare no conflict of interest.

## ETHICS STATEMENT

The university institutional review board and the hospital institutional review board approved this study.

## Supporting information

Supporting information.

## Data Availability

The data sets generated during and/or analyzed during the current study are not publicly available, but are available from the corresponding author on reasonable request.
