# Supplementary material for: The impact of professional role and demographic characteristics on job satisfaction and retention among healthcare professionals in a military hospital
Source: Nurs Forum. 2022 Jul 9;57(6):1034–43. doi: 10.1111/nuf.12777 (PMC10083962; doi:10.1111/nuf.12777)
Supplement: Supplementary file 1 — Supporting information. [file NUF-57-1034-s001.docx]

Supplementary Material File 1

**Military Healthcare Professionals Relational Coordination, Job Satisfaction,**

**and Retention Survey**

1. **Frequent Communication**

How **frequently** do people in each of these groups communicate with you about **patient care**?

*When answering this question, be sure to consider all forms of communication, including in-person*

*meetings, phone calls, e-mails, etc.*

***Select the Not Applicable (N/A) answer choice if interaction with the workgroup/individual listed is***

***not needed with your role or if you do not wish to answer this question.***

| **LPN’s (Licensed Practical Nurses)** | Not Nearly Enough   | Not  Enough   | Just the Right Amount   | Too Often   | Much Too Often   | Not  Applicable   |
| --- | --- | --- | --- | --- | --- | --- |
| **RN’s (Registered Nurses)** | Not Nearly Enough   | Not  Enough   | Just the Right Amount   | Too Often   | Much Too Often   | Not  Applicable   |
| **Residents** | Not Nearly Enough   | Not  Enough   | Just the Right Amount   | Too Often   | Much Too Often   | Not  Applicable   |
| **Physicians** | Not Nearly Enough   | Not  Enough   | Just the Right Amount   | Too Often   | Much Too Often   | Not  Applicable   |

1. **Timely Communication**

*Do they communicate with you in a* ***timely*** *way about* ***patient care****?*

*When answering this question, be sure to consider all forms of communication, including in-person*

*meetings, phone calls, e-mails, etc.*

***Select the Not Applicable (N/A) answer choice if interaction with the workgroup/individual listed is***

***not needed with your role or if you do not wish to answer this question.***

| **LPN’s (Licensed Practical Nurses)** | Never   | Rarely   | Sometimes   | Often   | Always   | Not  Applicable   |
| --- | --- | --- | --- | --- | --- | --- |
| **RN’s (Registered Nurses)** | Never   | Rarely   | Sometimes   | Often   | Always   | Not  Applicable   |
| **Residents** | Never   | Rarely   | Sometimes   | Often   | Always   | Not  Applicable   |
| **Physicians** | Never   | Rarely   | Sometimes   | Often   | Always   | Not  Applicable   |

©Relational Coordination Analytics, Inc. All rights reserved worldwide. RC Survey 2.0. 2013-2020

**Military Healthcare Professionals Relational Coordination, Job Satisfaction,**

**and Retention Survey**

1. **Accurate Communication**

Do they **communicate** with you *accurately* about **patient care**?

*When answering this question, be sure to consider all forms of communication, including in-person*

*meetings, phone calls, e-mails, etc.*

***Select the Not Applicable (N/A) answer choice if interaction with the workgroup/individual listed is***

***not needed with your role or if you do not wish to answer this question.***

| **LPN’s (Licensed Practical Nurses)** | Never   | Rarely   | Sometimes   | Often   | Always   | Not  Applicable   |
| --- | --- | --- | --- | --- | --- | --- |
| **RN’s (Registered Nurses)** | Never   | Rarely   | Sometimes   | Often   | Always   | Not  Applicable   |
| **Residents** | Never   | Rarely   | Sometimes   | Often   | Always   | Not  Applicable   |
| **Physicians** | Never   | Rarely   | Sometimes   | Often   | Always   | Not  Applicable   |

1. **Problem-Solving Communication**

When there is a problem with **patient care,** do people in each of these groups blame others or work with you to ***solve*** *the problem*?

*When answering this question, be sure to consider all forms of communication, including in-person*

*meetings, phone calls, e-mails, etc.*

***Select the Not Applicable (N/A) answer choice if interaction with the workgroup/individual listed is***

***not needed with your role or if you do not wish to answer this question.***

| **LPN’s (Licensed Practical Nurses)** | Always Blame   | Mostly Blame   | Neither Blame Nor Solve   | Mostly  Solve   | Always Solve   | Not  Applicable   |
| --- | --- | --- | --- | --- | --- | --- |
| **RN’s (Registered Nurses)** | Always Blame   | Mostly Blame   | Neither Blame Nor Solve   | Mostly  Solve   | Always Solve   | Not  Applicable   |
| **Residents** | Always Blame   | Mostly Blame   | Neither Blame Nor Solve   | Mostly  Solve   | Always  Solve   | Not  Applicable   |
| **Physicians** | Always Blame   | Mostly Blame   | Neither Blame Nor Solve   | Mostly  Solve   | Always Solve   | Not  Applicable   |

©Relational Coordination Analytics, Inc. All rights reserved worldwide. RC Survey 2.0. 2013-2020

**Military Healthcare Professionals Relational Coordination, Job Satisfaction,**

**and Retention Survey**

1. **Shared Goals**

Do people in each of these groups ***share your goals*** for **patient care**?

*When answering this question, be sure to consider all forms of communication, including in-person*

*meetings, phone calls, e-mails, etc.*

***Select the Not Applicable (N/A) answer choice if interaction with the workgroup/individual listed is***

***not needed with your role or if you do not wish to answer this question.***

| **LPN’s (Licensed Practical Nurses)** | Not At All   | A Little   | Somewhat   | A Lot   | Completely   | Not  Applicable   |
| --- | --- | --- | --- | --- | --- | --- |
| **RN’s (Registered Nurses)** | Not At All   | A Little   | Somewhat   | A Lot   | Completely   | Not  Applicable   |
| **Residents** | Not At All   | A Little   | Somewhat   | A Lot   | Completely   | Not  Applicable   |
| **Physicians** | Not At All   | A Little   | Somewhat   | A Lot   | Completely   | Not  Applicable   |

1. **Shared Knowledge**

Do people in each of these groups ***know*** about the work you do with **patient care**?

*When answering this question, be sure to consider all forms of communication, including in-person*

*meetings, phone calls, e-mails, etc.*

***Select the Not Applicable (N/A) answer choice if interaction with the workgroup/individual listed is***

***not needed with your role or if you do not wish to answer this question.***

| **LPN’s (Licensed Practical Nurses)** | Nothing   | A Little   | Some   | A Lot   | Everything   | Not  Applicable   |
| --- | --- | --- | --- | --- | --- | --- |
| **RN’s (Registered Nurses)** | Nothing   | A Little   | Some   | A Lot   | Everything   | Not  Applicable   |
| **Residents** | Nothing   | A Little   | Some   | A Lot   | Everything   | Not  Applicable   |
| **Physicians** | Nothing   | A Little   | Some   | A Lot   | Everything   | Not  Applicable   |

©Relational Coordination Analytics, Inc. All rights reserved worldwide. RC Survey 2.0. 2013-2020

**Military Healthcare Professionals Relational Coordination, Job Satisfaction,**

**and Retention Survey**

1. **Mutual Respect**

Do people in each of these groups ***respect*** the work you do with **patient care**?

*When answering this question, be sure to consider all forms of communication, including in-person*

*meetings, phone calls, e-mails, etc.*

***Select the Not Applicable (N/A) answer choice if interaction with the workgroup/individual listed is***

***not needed with your role or if you do not wish to answer this question.***

| **LPN’s (Licensed Practical Nurses)** | Not At All   | A Little   | Somewhat   | A Lot   | Completely   | Not  Applicable   |
| --- | --- | --- | --- | --- | --- | --- |
| **RN’s (Registered Nurses)** | Not At All   | A Little   | Somewhat   | A Lot   | Completely   | Not  Applicable   |
| **Residents** | Not At All   | A Little   | Somewhat   | A Lot   | Completely   | Not  Applicable   |
| **Physicians** | Not At All   | A Little   | Somewhat   | A Lot   | Completely   | Not  Applicable   |

***Please answer the following questions:***

1. Which role do you belong to?

| **LPN (Licensed Practical Nurse)** |  |
| --- | --- |
| **RN (Registered Nurse)** |  |
| **Resident** |  |
| **Physician** |  |

1. Which hospital unit do you work on?

©Relational Coordination Analytics, Inc. All rights reserved worldwide. RC Survey 2.0. 2013-2020

**Military Healthcare Professionals Relational Coordination, Job Satisfaction,**

**and Retention Survey**

**Intent to Stay Scale (Military Nurses, Residents, and Physicians)**

**THIS QUESTION IS FOR MILITARY HEALTHCARE PROFESSIONALS, PLEASE PROCEED TO THE NEXT PAGE IF YOU ARE A CIVILIAN HEALTHCARE PROFESSIONAL**.

***Please answer the following questions:***

1. I plan to leave the Army as soon possible.

| Strongly Agree   | Agree   | Neither Agree or Disagree   | Disagree   | Strongly Disagree   |
| --- | --- | --- | --- | --- |

1. Under no circumstances will I voluntarily leave the Army

| Strongly Agree   | Agree   | Neither Agree or Disagree   | Disagree   | Strongly Disagree   |
| --- | --- | --- | --- | --- |

1. I would be reluctant to leave the Army.

| Strongly Agree   | Agree   | Neither Agree or Disagree   | Disagree   | Strongly Disagree   |
| --- | --- | --- | --- | --- |

1. I plan to stay in the Army as long as possible.

| Strongly Agree   | Agree   | Neither Agree or Disagree   | Disagree   | Strongly Disagree   |
| --- | --- | --- | --- | --- |

©Relational Coordination Analytics, Inc. All rights reserved worldwide. RC Survey 2.0. 2013-2020

**Military Healthcare Professionals Relational Coordination, Job Satisfaction,**

**and Retention Survey**

**Intent to Stay Scale. (Civilian Nurses, Residents, and Physicians)**

**THIS QUESTION IS FOR CIVILIAN HEALTHCARE PROFESSIONALS, PLEASE PROCEED TO THE NEXT PAGE IF YOU ARE A MILITARY HEALTHCARE PROFESSIONAL.**

***Please answer the following questions:***

1. I plan to leave this hospital as soon possible.

| Strongly Agree   | Agree   | Neither Agree or Disagree   | Disagree   | Strongly Disagree   |
| --- | --- | --- | --- | --- |

1. Under no circumstances will I voluntarily leave this hospital.

| Strongly Agree   | Agree   | Neither Agree or Disagree   | Disagree   | Strongly Disagree   |
| --- | --- | --- | --- | --- |

1. I will be reluctant to leave this hospital.

| Strongly Agree   | Agree   | Neither Agree or Disagree   | Disagree   | Strongly Disagree   |
| --- | --- | --- | --- | --- |

1. I plan to stay at this hospital as long as possible.

| Strongly Agree   | Agree   | Neither Agree or Disagree   | Disagree   | Strongly Disagree   |
| --- | --- | --- | --- | --- |

©Relational Coordination Analytics, Inc. All rights reserved worldwide. RC Survey 2.0. 2013-2020

**Military Healthcare Professionals Relational Coordination, Job Satisfaction,**

**and Retention Survey**

**Job Satisfaction**

***Please answer the following questions:***

1. On the whole, how satisfied are you with your present job?

| Very Dissatisfied   | Dissatisfied   | Neutral   | Satisfied   | Very Satisfied   |
| --- | --- | --- | --- | --- |

**Demographic Questions**

***Please answer the following questions:***

1. What is your age in years?
2. What is your race?

| Asian   | Black/African   | American Indian   | Caucasian   | Hispanic   | Other   |
| --- | --- | --- | --- | --- | --- |

1. What is your sex?

| Female   | Male   |
| --- | --- |

©Relational Coordination Analytics, Inc. All rights reserved worldwide. RC Survey 2.0. 2013-2020

**Military Healthcare Professionals Relational Coordination, Job Satisfaction,**

**and Retention Survey**

**Demographic Questions**

***Please answer the following questions:***

1. Military Officers: What is your rank?

| O-1   | O-2   | O-3   | O-4   | O-5   | O-6   |
| --- | --- | --- | --- | --- | --- |

1. Military Enlisted: What is your rank?
2. Federal or Civilian Employees: What is you General Schedule (GS) rate? Please list your GS rate if your GS rate is not listed below.

| GS-8   | GS-9   | GS-10   | GS-11   | GS-12   | GS-13   |
| --- | --- | --- | --- | --- | --- |

1. What is your highest completed degree?

| Diploma   | Associate Degree   | Baccalaureate Degree   | Graduate Degree   |
| --- | --- | --- | --- |

1. Please list any credentials you have related to your job role.
2. How long have you been in your career (e.g., How long have you been a nurse or physician)?
3. How long have you worked at this hospital?
4. How long have you worked on this unit?

©Relational Coordination Analytics, Inc. All rights reserved worldwide. RC Survey 2.0. 2013-2020
